# 

**Published:** 1812-05

**Authors:** 


### MONTHLY CATALOGUE OF MEDICAL BOOKS.

NATURAL History, General and Particular, by the Count de Buffon, illustrated with above six hundred plates.—The History of Man and Quadrupeds translated, with notes and observations. By William Smellie, Member of the Antiquarian and Royal Societies of Edinburgh.—A new edition, carefully corrected, and considerably enlarged by many additional articles, notes, and plates, and some account of the Life of M. de Buffon. By William Wood, F.L.S. In 20 vols. 8vo.—Cadell and Davies.

Anatomical Examinations.—A complete Series of Anatomical Questions, with Answers; the answers arranged so as to form an elementary system of anatomy, and intended as preparatory to examinations at Surgeons' Hall. To which are annexed, Tables of the Bones, Muscles, and Arteries. The second edition, corrected, in 2 vols.—Highley.

A View of the Nervous Temperament, being a Practical Inquiry in the increasing Prevalence, Prevention, and Treatment, of those Diseases commonly called Nervous, Bilious, Stomach, and Liver, Complaints; Indigestion; Low Spirits; Gout, &c. By T. Trotter, M.D. The third edition, with large additions.

---

### *NOTICES TO CORRESPONDENTS.*

*A very respectable physician, and friend to the Medical Journal, has requested to know upon what authority Dr. William Harvey is placed among the practitioners in midwifery in 1653, as stated in the Collectanea, p. 117. Having received that fact from an intelligent and investigating physician, we hope that he will satisfy the inquirer.*

*We presume Justitia intended his remarks for some other periodical work; being neither medical nor physical, they cannot with any propriety appear in our Journal. If Mr. Saumarez has inserted in his new work "on the principles of Physiological and Physical Science," a note copied verbatim from Bishop Horseley's nineteenth sermon, explaining the different import of paradox and contradiction, he will, we doubt not, at the instance of Justitia, make the proper acknowledgment.*

*Having mislaid the paper containing the Resolutions of the Medical Practitioners at Kendal, we must refer Mr. Burne to any of the gentlemen who signed the resolutions, and whose names are inserted in the Medical Journal for January, p. 54.*

*We are obliged to J. M. B. Pigot, M.D.; and to Messrs. Green, Phythian, Hastings, Parish, Medicus, &c. &c. for their respective Communications, which will be published in our next Number.*

